# Attitudes toward and exposure to gender discrimination in work life by pulmonologists and thoracic surgeons: a questionnaire-based survey among Turkish thoracic society members

**DOI:** 10.3389/fmed.2024.1463732

**Published:** 2024-11-20

**Authors:** Birsen Ocakli, Arzu Yorgancioglu, Sinem Gungor, Fusun Topcu, Yesim Yigiter Senol, Basak Goktas, Nurdan Kokturk, Eylem Tuncay, Baran Gundogus, Hilal Altinoz, Yesim Yasin, Ipek Ozmen, Serap Duru, Elif Ozari Yildirim, Tulin Sevim, Cansel Atinkaya Ozturk, Esra Uzaslan, the Turkish Thoracic Society Women and Lung Diseases Working Group

**Affiliations:** ^1^Clinic of Chest Diseases, University of Health Sciences Istanbul Sureyyapasa Chest Diseases and Thoracic Surgery Training and Research Hospital, Istanbul, Türkiye; ^2^Department of Chest Diseases, Celal Bayar University Faculty of Medicine, Manisa, Türkiye; ^3^Department of Chest Diseases, Dicle University Faculty of Medicine, Diyarbakir, Türkiye; ^4^Department of Public Health, Akdeniz University Faculty of Medicine, Antalya, Türkiye; ^5^Department of Chest Diseases, Gazi University Faculty of Medicine, Ankara, Türkiye; ^6^Department of Public Health, Acibadem University Faculty of Medicine, Istanbul, Türkiye; ^7^Clinic of Chest Diseases and Thoracic Surgery, University of Health Sciences Ankara Yildirim Beyazit Training and Research Hospital, Ankara, Türkiye; ^8^Department of Chest Diseases, Uludag University Faculty of Medicine, Bursa, Türkiye

**Keywords:** gender discrimination, physicians, chest diseases, thoracic surgery, gender roles

## Abstract

**Background:**

This study aimed to evaluate attitudes toward and exposure to gender discrimination in work life by chest diseases specialists and thoracic surgeons.

**Methods:**

A total of 275 members of Turkish Thoracic Society (TTS) were included on a voluntary basis in this online cross-sectional questionnaire-survey using an internal member-only social media platform of TTS. The questionnaire form elicited items on sociodemographic characteristics, occupational characteristics and gender discrimination in work life (general opinions, attitudes and exposure).

**Results:**

Female doctors (vs. males) were less likely to be a thoracic surgeon (13.8% vs. 34.5%, *p* < 0.05) and a professor of thoracic surgery (0.0% vs. 26.7% vs. *p* < 0.05), and more likely to consider housework as a considerable burden (89.8 vs. 73.6%, *p* = 0.02) and the significant role of discriminatory, negative and dissuasive attitudes of male physicians in their career choice (67.6 vs. 35.6%, *p* = 0.039). Male doctors were more likely to considered that men are more successful in specialties that require active physical strength (65.5 vs. 27.7%, *p* = 0.005) and those with very long working hours and heavy shifts (57.5 vs. 39.4%, *p* = 0.001). Female thoracic surgeons were more likely than males to consider that specialties with very long working hours and heavy shifts are more suitable for men (26.9 vs. 6.0%, *p* = 0.027) and men are given priority in academic career promotion (64.0 vs. 13.3%, *p* < 0.001). Younger (vs. older) females reported higher rate of exposure to gender discrimination (*p* = 0.041) and considerable impact of social roles on the specialty (*p* = 0.007), while female doctors working as a resident (33.8%) and a specialist (50.05%) indicated higher rate of exposure to gender discrimination during their career (*p* = 0.024).

**Conclusion:**

In conclusion, our findings revealed that exposure to gender discrimination in work life was more commonly expressed by female members of TTS, particularly in terms of burden of social roles, career advancement options and leadership positions, along with significant role of discriminatory, negative and dissuasive attitudes of male physicians in their career choice. Accordingly, women remain underrepresented in thoracic surgery, particularly in the academic rank of full professor and in leadership positions with inability to promote after a definite step in their careers.

## Introduction

Gender discrimination has structural, social and cultural dimensions overall, while in the healthcare workplace it is also contradictory to the principles of professionalism and results in marked and far-reaching adverse effects on the health care workforce, delivery of patient care, and advancement of science (1, 2). Increasing representation of women in medicine reflects the profession’s commitment toward inclusivity and diversity to improve patient care and system efficacy (3). Beyond simply joining the workforce, achieving equitable positions of leadership and influence is also critical to engage systematic change and address bias throughout local, regional, and national levels (3, 4).

However, women in medicine continue to face gender discrimination despite the fact that they are highly educated and qualified members comprising significant portion of the active physician workforce (1, 5, 6). The underrepresentation of female physicians in leadership roles and in certain specialties, particularly in the surgery field results in lower number of women surgeons as compared to other specialties to practice world-wide, despite relatively similar numbers of women and men graduating from medical school (2, 5, 7). In Turkey, while recruitment of candidate doctors to medical specializations was based on the application of as a centrally-graded Examination of Specialization in Medicine (ESM) for more than 30 years, enabling female or male doctors to enter their department of choice if they have the required grades, the number of women is still limited especially in surgery specialization fields (8). Surgical personality and culture, sexism, lifestyle and workload are considered among the potential deterrents for women considering a surgical career (9).

Among the surgical specialties, thoracic surgery has been a male-dominated medical specialty since its inception which still exhibits gender disparities across multiple domains (i.e., recruitment, career progression, academic promotion, salary gaps and professional development opportunities), despite positive trends in other surgical subspecialties (4, 10–12).

According to data from Association of American Medical Colleges in (13), women only comprised 8% (369 out of 4,448) of active thoracic surgeons, compared with the 37% (351,117 out of 946,790) seen across all medical specialties (13). In a study from Turkey assessing the 28 fields of medical specialization, the rate of female doctors in surgery specialization fields was reported to be below 33% along with the higher rate of feeling discrimination by female doctors in the fields of surgery than other disciplines, while the rate of the male doctors was not under 33% in any of the 28 specialization fields (8). Hence, women’s presence in thoracic surgery appeared to be below the critical mass of 30% identified as a threshold for a group to achieve substantive representation of its interests (14, 15), which seems notable given that gender-based disparities in thoracic surgery can be difficult to recognize without transparency, reporting, or reflection (4, 8).

Despite the improved representation of women in medicine, there remains a noticeable disparity in leadership roles and opportunities of academic advancement for women in this field, despite the fact that female doctors are perceived as more alert with a greater tendency to stick to clinical guidelines thus being more likely to deliver evidence-based treatment and better communication with patients and families (9, 11, 16). Besides the bias in allocating prestigious positions, promotion and sponsorship, there are persistent organizational barriers (i.e., female-unfriendly work environment and culture of gender discrimination in surgery) which create a ‘glass ceiling’ that prevents female doctors from rising beyond a certain level in the surgery hierarchy (4, 17). Hence, it remains unclear whether the increased volume of female thoracic surgery trainees and practicing surgeons have been accompanied by similar advances in opportunities for academic achievement and leadership roles within academic organizations (4, 18).

Hence, addressing the gender discrimination in healthcare workplace is necessary to understand working conditions of female physicians and to recognize the existence and magnitude of gender discrimination (2, 5, 19). This is particularly important for traditionally male-dominated fields such as thoracic surgery, where women receive less personal support and career advancement and are often disregarded professionally by patients and other physicians (2, 5, 10, 19, 20).

This cross-sectional questionnaire-based survey was therefore designed to evaluate the prevalence and nature of gender discrimination among pulmonologists and thoracic surgeons who are members of the Turkish Thoracic Society (TTS), by addressing their opinions and attitudes toward gender discrimination as well as their exposure to gender discrimination in work life in relation to potential sociodemographic correlates.

## Materials and methods

### Study population

The Turkish Thoracic Society involves 6,469 members including 3,750 (58%) males and 2,719 (42%) females across all membership type. A total of 3,454 members of TTS were invited to participate in this online cross-sectional questionnaire-survey via completing the questionnaire form provided through an internal member-only social media platform of TTS on a voluntary basis and 275 physicians (188 females and 87 males) who completed the online questionnaire form were included in the study conducted between January 2020 and February 2020. The survey was open for 30 days, while reminder emails sent to nonrespondents to remind them to participate in the survey before its closing.

Written informed consent was obtained from each subject following a detailed explanation of the objectives and protocol of the study which was conducted in accordance with the ethical principles stated in the “Declaration of Helsinki” and approved by the University of Health Sciences, Sureyyapasa Chest Diseases and Thoracic Surgery Training and Research Hospital Ethics Committee (Date of Approval: 22/04/2020, Protocol No: 094).

### The questionnaire and study parameters

The questionnaire form elicited items on sociodemographic characteristics (age, gender, marital status, number of children, partner’s occupation), occupational characteristics (hospital type, specialty, academic title) and gender discrimination in work life (general opinions, attitudes and exposure). The items related to gender discrimination in work life were adapted from a 24-item gender discrimination questionnaire developed for a medical specialty (Public Health) thesis on examination the gender roles attitudes of residencies and their exposure to gender discrimination by qualitative and quantitative methods in Turkey (21). Each item in the subscales of general opinions and attitudes toward gender discrimination (12 items) and exposure to gender discrimination in work life (10 items) was evaluated via a 5-point Likert scale (ranges from 1 [strongly disagree] to 5 [strongly agree]). The form was completed by each volunteer online. Gender discrimination parameters were evaluated with respect to physicians’ sociodemographic and occupational characteristics (Supplementary file).

### Statistical analysis

Statistical analysis was made using IBM SPSS Statistics for Windows, version 22.0 (IBM Corp., Armonk, NY). Pearson Chi-square (χ^2^) test (Yates Correction or Fisher’s Freeman Halton Exact Test with *post-hoc* Bonferroni correction where available) was used for the comparison of categorical data. Data were expressed as percent (%). *p* < 0.05 was considered statistically significant.

## Results

### Sociodemographic and occupational characteristics

Overall, 275 out of 3,454 members of TTS who were invited to participate in the study completed the online questionnaire form with a response rate of 8.0%. In total, 66.2% of participants aged <45 years and 68.4% were females, while 77.5% were married (to another physician: 48.4%), and 63.6% had a child. Most of participants were working in chest diseases clinic (70.2%) at university hospitals (40.4%) or training and research hospitals (35.6%) as specialists (43.3%; Table 1).

**Table 1:** Sociodemographic and occupational characteristics.

	Total (*n* = 275)	Male (*n* = 87)		Female (*n* = 188)		*p* value
Gender, n(%)						
Female	188 (68.4)	–		–		–
Male	87 (31.6)	–		–		
Age group, n(%)						
25–34 y	92 (33.5)	19 (21.8)		73 (38.8)^*^		**0.027**
35-44 y	90 (32.7)	30 (34.5)		60 (31.9)		
45–54 y	60 (21.8)	25 (28.7)		35 (18.6)		
55–64 y	30 (10.9)	11 (12.6)		19 (10.1)		
≥65 y	3 (1.1)	2 (2.3)		1 (0.5)		
Marital status, n(%)						
Married	213 (77.5)	78 (89.7)		135 (71.8)^*^		**0.002**
Single	59 (21.5)	9 (10.3)		50 (26.6)^*^		
Divorced	3 (1.1)	0 (0)		3 (1.6)		
Children number, n(%)						
None	100 (36.4)	14 (16.1)		86 (45.7)^*^		**<0.001**
1	84 (30.6)	35 (40.2)		49 (26.1)^*^		
2	81 (29.5)	32 (36.8)		49 (26.1)		
≥3	10 (3.6)	6 (6.9)		4 (2.1)^*^		
Partner’s occupation, n(%)						
No partner	43 (15.6)	4 (4.6)		41 (21.8)^*^		**<0.001**
Unemployed	12 (4.4)	7 (8.0)		5 (2.7)^*^		
Physician	133 (48.4)	42 (48.3)		91 (48.4)		
Other	85 (30.9)	34 (39.1)		51 (27.1)^*^		
Hospital type, n(%)						
University hospital	111 (40.4)	30 (34.5)		81 (43.1)		0.464
State hospital	37 (13.5)	12 (13.8)		25 (13.3)		
Training and research hospital	98 (35.6)	33 (37.9)		65 (34.6)		
Other	29 (10.6)	12 (13.8)		17 (9)		
Specialty, n(%)						
Pulmonologist	193 (70.2)	54 (62.1)		139 (73.9)^*^		**<0.001**
Thoracic surgeon	56 (20.4)	30 (34.5)		26 (13.8)^*^		
Other^a^	26 (9.5)	3 (3.4)		23 (12.2)^*^		
Academic title, n(%)						
Resident	69 (25.1)	19 (21.8)		50 (26.6)		0.747
Specialist	119 (43.3)	36 (41.4)		83 (44.1)		
Asst. Prof.	11 (4.0)	3 (3.4)		8 (4.3)		
Assoc. Prof.	28 (10.2)	10 (11.5)		18 (9.6)		
Prof.	36 (13.1)	15 (17.2)		21 (11.2)		
Other	12 (4.4)	4 (4.6)		8 (4.3)		
Academic title by specialty-gender, n(%)		Pulm (*n* = 54)	Surg (*n* = 30)	Pulm (*n* = 139)	Surg (*n* = 26)	
Resident		12 (22.2)	7 (23.3)	35 (25.2)	10 (38.5)	**0.035**
Specialist		24 (44.4)	10 (33.3)	60 (43.2)	11 (42.3)	
Assistant Professor		1 (1.9)	1 (3.3)	7 (5.0)	1 (3.8)	
Associate Professor		7 (13.0)	3 (10.0)	13 (9.4)	3 (11.5)	
Professor		7 (13.0)	8 (26.7)	21 (15.1)	0 (0.0)	
Other		3 (5.5)	1 (3.3)	3 (2.2)	1 (3.8)	

a resident, general practitioner, instructor, family physician. Pulm, Pulmonologist; Surg, Surgeon.

Pearson Chi-Square Test, Fisher’s Freeman Halton Exact Test, Post-Hoc Bonferroni Correction. *p < 0.05 compared to males. Values in bold indicate statistical significance (*p* < 0.05).

Female participants were more likely to be in the 25–34 y (38.8% vs. 21.8%, *p* < 0.05) age group, and to be single (26.6% vs. 10.3%, *p* < 0.05) with no child (45.7% vs. 16.1%, *p* < 0.05) Also, females were more likely to be pulmonology specialists (73.9% vs. 62.1%, *p* < 0.05) rather than thoracic surgeons (13.8% vs. 34.5%, *p* < 0.05), when compared to males. No significant difference was noted between female and male physicians overall in terms the academic title (Table 1).

However, when analyzed with respect to specialties, professor rates in thoracic surgery were significantly higher among males vs. females (26.7% vs. 0.0%, *p* < 0.05), while professor rates in pulmonology were similar between males and females (13.0 and 15.1%; Table 1).

Of 26 female thoracic surgeons participated in the study, most were working as resident (38.5%) or specialist (42.3%), while only 3.8% were assistant professors, 11.5% were associates, and none were full professors (Table 1).

### General opinions and attitudes toward gender discrimination

Considering general opinions and attitudes toward gender discrimination, significantly higher percentage of females vs. males agreed/strongly agreed that housework is a considerable burden for female doctors (89.8 vs. 73.6%, *p* = 0.02), female physicians are more exposed to discrimination because of their clothes (54.5 vs. 36.8%, *p* = 0.007) and to disturbing behavior of the opposite sex in their professional life (73.4 vs. 54.0%, *p* = 0.007), the discriminatory, negative and dissuasive attitudes of male physicians played a role in the female physicians not choosing surgical branches (67.6 vs. 35.6%, *p* = 0.039), and women physicians are more successful in passive jobs that require patience and punctuality (57.4 vs. 14.9%, *p* = 0.001). However, significantly higher percentage of males vs. females agreed/strongly agreed that men are more successful in specialties that require active physical strength (65.5 vs. 27.7%, *p* = 0.005), specialties with very long working hours and heavy shifts are more suitable for men (57.5 vs. 39.4%, *p* = 0.001) and female physicians cannot succeed in surgical branches (14.9 vs. 2.1%, *p* = 0.001; Table 2).

**Table 2:** Physicians’ opinions, attitudes and experience regarding gender discrimination by gender.

	Percentage of respondents who agreed/strongly agreed on items			
	Total	Male	Female	*p* value
General opinions and attitudes toward gender discrimination (Items 1–12)	%	%	%	
The sex of physician should be a criterion to be considered during provider selection	6.2	9.2	4.8	>0.05
Besides work in the workplace, housework is also an important burden for the female physician.	84.7	73.6	89.8	**0.02**
If a sacrifice is to be made between spouses for a career in working life, this task falls on woman.	12.8	8.0	15.0	>0.05
Women physicians are exposed to more discrimination because of their clothes.	48.9	36.8	54.5	**0.007**
Social roles (motherhood, housework) are largely influential on the specialty choices of female physicians	69.7	66.7	71.1	>0.05
Women physicians are more successful in passive jobs that require patience and punctuality.	44.0	14.9	57.4	**0.001**
Men are more successful in specialties that require active physical strength (surgery, etc.)	39.6	65.5	27.7	**0.005**
Specialties with very long working hours and heavy shifts are more suitable for men	45.1	57.5	39.4	**0.001**
Female physicians cannot succeed in surgical branches	6.2	14.9	2.1	**0.001**
Patients trust male doctors more and take them seriously	51.1	44.8	54.0	>0.05
I think that the discriminatory, negative and dissuasive attitudes of male physicians played a role in the female physicians not choosing surgical branches.	57.5	35.6	67.6	**0.039**
Female physicians are more exposed to the disturbing behavior of the opposite sex in their professional life compared to male physicians due to their gender.	67.3	54.0	73.4	**0.007**
Exposure to gender discrimination in work life (Items 13–22)	%	%	%	
My gender had an impact on my choice of the department I am currently in	23.6	12.6	28.7	>0.05
I have been exposed to gender discrimination throughout my career	34.3	16.1	42.6	**0.001**
While performing my profession, my gender negatively affected my working life	32.2	12.6	41.0	**0.001**
In the unit where I work, men are given priority in academic career promotion	17.2	6.9	22.0	**0.003**
In the unit where I work, work-sharing is based on the order of precedence regardless of the gender	72.7	79.1	69.7	>0.05
In the unit where I work participation in decision making (getting opinions etc.) does not depend on gender	79.2	89.7	74.3	**0.038**
In the unit I work in, sexist expressions such as “try do a man’s job and “women’s reason” are used in conversations and discussions	11.7	5.7	14.5	**0.001**
The facilities of the department I work in (education, participation to congress etc.) are used without any gender discrimination	85.0	88.5	83.4	>0.05
In the unit where I work, opinions of men rather than women are considered despite their same order of precedence	13.9	3.4	18.8	**0.005**
In the unit where I work, mostly male physicians are preferred due to the reasons such as pregnancy and breast-feeding permissions.	40.4	31.0	44.4	**0.001**

χ^2^ test. Values in bold indicate statistical significance (*p* < 0.05).

### Exposure to gender discrimination in work life

Significant higher percentage of females vs. males agreed/strongly agreed that they are exposed to gender discrimination throughout their career (42.6 vs. 16.1%, *p* = 0.001) negatively affecting their professional life (41.0 vs. 12.6%, *p* = 0.001), men are given priority in academic career promotion (22.0 vs. 6.9%, *p* = 0.003) and in employment (44.4 vs. 31.0%, *p* = 0.001) and their opinions are more respected (18.8 vs. 3.4%, *p* = 0.005), and use of sexist expressions during conversations and discussions are common in their department (14.5 vs. 5.7%, *p* = 0.001; Table 2).

Significantly higher percentage of males vs. females agreed/strongly agreed that participation in decision making is independent of gender in their department (89.7% vs. 74.3%, *p* = 0.038; Table 2).

### Gender discrimination by specialty, age and academic title

In both pulmonology and thoracic surgery, males were more likely than females to consider that men are more successful in specialties that require active physical strength (64.8 vs. 34.5%, *p* < 0.001 and 63.3 vs. 15.4%, *p* = 0.001, respectively), while females were more likely than males to consider that the discriminatory, negative and dissuasive attitudes of male physicians played a role in the female physicians not choosing surgical branches (61.9 vs. 35.2%, *p* = 0.001 and 84.6 vs. 3.0%, *p* < 0.001, respectively) and opinions of men rather than women are more respected despite their same title in their department (13.0 vs. 1.9%, *p* = 0.039 and 52.0 vs. 6.7%, *p* < 0.001, respectively; Table 3).

**Table 3:** Physicians’ opinions, attitudes and experience regarding gender discrimination by gender across specialty, age and academic title.

	Male and female respondents who agreed/strongly agreed on items according to specialty, %							
	Pulmonologists				Thoracic surgeons			
	n	Males	Females	*p*	n	Males	Females	*p*
Questionnaire items with significance								
Men are more successful in specialties that require active physical strength (i.e., surgery)	193	64.8	34.5	**<0.001**	56	63.3	15.4	**0.001**
Specialties with very long working hours and heavy shifts are more suitable for men	193	55.6	46.0	0.235	56	6.0	26.9	**0.027**
The discriminatory, negative and dissuasive attitudes of male physicians play a role in the female physicians not choosing surgical branches.	193	35.2	61.9	**0.001**	56	3.0	84.6	**<0.001**
Men are given priority in academic career promotion in my department	192	3.7	14.5	0.063	55	13.3	64.0	**<0.001**
Participation in decision making does not depend on gender in my department	193	90.7	80.6	0.136	55	90.0	36.0	**<0.001**
Opinions of men rather than women are more respected despite their same title in my department	192	1.9	13.0	**0.039**	55	6.7	52.0	**<0.001**

	Male and female respondents who agreed/strongly agreed on items according to age, %													
	Males							Females						
	n	25–34	35–44	45–54	55–64	≥65	*p*	n	25–34	35–44	45–54	55–64	≥65	*p*
Questionnaire items with significance														
I have been exposed to gender discrimination throughout my career	14	14.3	50.0	28.6	0	7.1	0.247	80	51.2	25.0	15.0	8.8	0.0	**0.041**
Social roles are largely influential on the specialty-related choices of female physicians	58	25.9	36.2	25.9	8.6	3.4	0.271	133	38.8	31.9	18.6	10.1	0.5	**0.007**
Patients trust male doctors more and take them seriously	39	25.6	46.2	20.5	7.7	0.0	0.095	101	50.5	31.7	10.9	6.9	0	**0.001**

	Male and female respondents who agreed/strongly agreed on items according to academic title, %													
	Males							Females						
	Res	Spec	AsstP	AssocP	Prof.	Other	*p*	Res	Spec	AsstP	AssocP	Prof.	Other	*p*
Questionnaire items with significance														
I have been exposed to gender discrimination throughout my career	14.3	64.3	0	7.1	7.1	7.1	0.463	33.8	50.0	1.2	5.0	6.2	3.8	**0.025**
While performing my profession, my gender negatively affected my working life	0	81.8	9.1	0.0	9.1	0.0	**0.044**	23.4	54.5	1.3	10.4	5.2	**5.2**	**0.024**

CD, Chest diseases; TS, Thoracic surgery; Res, resident; Spec, Specialist; AsstP, Assistant Prof; AssocP, Associate ProfPearson Chi-Square Test, Yates Correction. Values in bold indicate statistical significance (*p* < 0.05).

Male thoracic surgeons vs. female thoracic surgeons were more likely to consider that participation in decision making is independent of gender in their department (90.0 vs. 36.0%, *p* < 0.001), while female thoracic surgeons were more likely than males to consider that specialties with very long working hours and heavy shifts are more suitable for men (26.9 vs. 6.0%, *p* = 0.027) and men are given priority in academic career promotion (64.0 vs. 13.3%, *p* < 0.001; Table 3).

No significant difference was noted among male physician by age, whereas younger age females including those aged 25–34 years and 35–44 years reported higher rate of exposure to gender discrimination throughout their career (51.2 and 25.0%, respectively, *p* = 0.041) and were more likely to consider the impact of social roles on the specialty choice of female physicians (38.8 and 31.9%, respectively, *p* = 0.007) and the patients to trust male doctors more and take them seriously (50.5 and 31.7%, respectively, *p* = 0.001; Table 3).

Considering academic title, specialists as compared with others were more likely to consider that their gender to negatively affect their professional life both among males (81.8%, *p* = 0.044) and females (54.5%, *p* = 0.024), while both resident (33.8%) and specialist (50.05%) females indicated higher rate of exposure to gender discrimination during their career than physicians with other academic titles (*p* = 0.024; Table 3).

## Discussion

Our findings in a cohort of pulmonologists and thoracic surgeons who are members of TTS revealed that female doctors were mainly working as pulmonologists rather than thoracic surgeons, along with lower rates of female professors particularly in the thoracic surgery where most female doctors were working as a resident or a specialist. Overall, there was a significant gender influence on doctors’ exposure to and opinions toward gender discrimination. Female doctors more commonly expressed that exposure to gender discrimination in work life had negative impact on their career with men given priority in academic career promotion and employment, while discriminatory, and negative and dissuasive attitudes of male physicians has a significant role in less likely preference of surgical branches by female doctors. Male doctors more commonly stated higher success of men in specialties that require active physical strength and those with very long working hours and heavy shifts and lower success of female physicians in surgical branches by male doctors.

Similarly, previous studies in various settings including surgery also indicated higher likelihood of female doctors to report gender discrimination and to feel their gender as a limitation that negatively affect their professional life (22–26). In a UK survey of women in surgery of all specialties, 88% of participants felt surgery was still male dominated, 59% reported or witnessed discrimination against women in the workplace, and that there was a lack of flexibility with 34% feeling that the profession was not conducive to motherhood (24). Notably, in a study among 45 female surgical trainees, female trainees in male-dominant specialties reported gender discrimination more frequently and as more severe and stressful experiences than those in female-dominant specialties (25). In a study among 156 female otorhinolaryngologists in Turkey, gender discrimination was reported to be 2.5 fold higher in departments where there were no female faculty members, while 53.2% of the female surgeons were reported to encounter gender discrimination during their residency programs (26).

Majority of our participants, regardless of gender, agreed on the burden of housework in the life of female physicians along with the considerable influence of social roles (motherhood, housework) on the specialty choices made by female physicians. Also, at least half of female doctors agreed that male physicians are preferred in their department due to concerns about pregnancy and breast-feeding permissions. These findings emphasize the significant contribution of traditional family/social roles and unwieldy maternity-leave policies in the gender discrimination reported in our cohort. Similarly, the greatest perceived barrier to women wanting to pursue and persist with a career in surgery was reported to be incongruity with motherhood and childcare commitments, since the surgical specialty often requires a working environment with patient treatment of unknown length or at unsocial times of day or night (24, 27), increasing the risk of excessive working hours, workload and conflicts with family commitments (28, 29). Moreover, female thoracic surgeons in our study specifically stated that specialties with very long working hours and heavy shifts are more suitable for men and men are given priority in academic career promotion. This supports the consideration of longer training hours and issues relating to work, family, and lifestyle as well as the frequently perceived role strain between family and professional life by female physicians to be the primary causes keeping women from entering surgical fields, despite they have equal interest in general surgery as males (7). Indeed, challenges for women entering thoracic surgery occur at all points along the pipeline, while concerns about lifestyle, lack of role models and mentorship, and a general culture not supportive to women in the specialty are suggested to contribute to discouraging women from entering the field (16, 30).

Although most of male pulmonologists and thoracic surgeons in our study agreed on higher success of men in specialties that require active physical strength (i.e., surgery) or those with very long working hours and heavy shifts, and considered social roles (i.e., motherhood, housework) to be largely influential on the specialty choices of female physicians, only one third of male pulmonologists and 3% of male thoracic surgeons agreed that the discriminatory, negative and dissuasive attitudes of male physicians play a role in the female physicians not choosing surgical branches. Indeed, bias, both conscious and unconscious, is considered to play a role, given that male surgeons are reported to perceived the milieu as more supportive of women than women did, while they are also less likely to agree that surgery is a good career for women (16, 31, 32).

Hence, our findings also emphasize not only the need for a change of behavior and attitude and improved supportive role among male surgeon colleagues but also a need that women to step up and promote themselves to break the glass ceiling for women in science (24, 33).Notably, female physicians in our cohort reported higher rate of exposure to gender discrimination throughout their career but particularly at younger ages during their initial years in practice. They were also more likely to consider the impact of social roles on the specialty choice of female physicians and believe that the patients trust male doctors more and take them seriously. Previous studies also indicated that junior women physicians were more vulnerable to gender discrimination and pressure to excel at work as competent doctors and mothers (34), as well as insufficient surgical confidence among female residents in terms of self-identifying themselves as a “surgeon” along with feeling their professional role to be disregarded more often by patients and physicians (9, 35). Higher exposure to gender discrimination at younger ages seems notable given the previous reports indicated that women have made more gains at the junior compared with senior level in cardiothoracic surgery and other subspecialties (18, 36). Although female residents in thoracic surgery was reported to be equally satisfied with career choice and to have a similar number of interviews and job offers compared with male residents, they felt less prepared technically and for practicing independently (16, 37). Another factor seems to be the inadequate mentoring with availability of few female mentors during formative years, as claimed to be a disadvantage by female surgeons especially in junior positions (17, 18, 38). The experience of higher gender discrimination by female surgeons early on in their career as compared to later may also be related to the possibility of them accepting gender discrimination later on in their lives.

Nonetheless, the dynamics of cultural and social practices constantly and implicitly recreate mechanisms to maintain gender inequality in academic medicine creating a vicious cycle that challenges the women physicians’ career development and causing gender-biased underemployment and poor career advancement to still exist in surgical fields (34, 39). Female surgeons are less likely to become board certified and more likely to experience burnout highlighting the challenges for women during training, while they also face challenges as they build a practice such as being less likely referred to by physicians and to be more likely to experience retributions after a complication than male surgeons, despite females demonstrating better outcomes overall (16, 40, 41). Moreover, the difficulties continue throughout their career, with women not progressing to leadership positions, remaining significantly underrepresented among editorial boards and in conferences (9, 42, 43). Interestingly, it has been reported that there have been noticeable increases in the representation of women on the editorial boards of high-impact cardiothoracic surgery journals, surpassing the percentage of women in the active US thoracic surgery workforce by nearly double (4). This trend could also reflect a response to workplace discrimination where women may feel compelled to exceed expectations for equitable recognition or may be another example of academic women surgeons putting in more time and effort toward work that does not appropriately translate into career advancement (4, 44).

However, discordance between perception and reality of the field has also been emphasized in surgical disciplines, given that female surgeons report a more positive view of their career choice and satisfaction at comparable rates to other specialties (7). Notably, in our study, female thoracic surgeons complained more than female pulmonologists regarding the negative influence of gender discrimination in choosing surgical branches, academic career promotion and reputability, while they were less troubled by very long working hours and heavy shifts, supporting that women within the surgical field report a satisfactory degree of control over their lifestyle (32). Notably, the culture of sexism has been considered to result in physical and social adaptations to fit into the role of surgeon, while significant effort to sustain this level of adaptation is considered likely to result in fatigue and creation of resilience mechanisms (25). The barriers may result in a selection of women with higher standards to gain entrance into the surgical workforce than men, which leads them to strive to be incredibly conscientious and dedicated, putting excess pressure on themselves (9). In fact, women who experienced negative gender bias were reported to have similar productivity but lower career satisfaction and self-efficacy scores than those without such experience in medical academic life, emphasizing the risk of deterioration in social and emotional well-being due to gender discrimination (45, 46).

In our study, females vs. males were significantly more likely to be pulmonologists rather than thoracic surgeons, while none of the female thoracic surgeons were professors and majority were working as a resident or specialist. Notably, disadvantages regarding the priority in academic career promotion and reputability were reported more prevalently by female thoracic surgeons than by male thoracic surgeons or female pulmonologists in our study. In this regard, our findings support that women remain underrepresented in thoracic surgery, particularly in the academic rank of full professor and in leadership positions, and they are unable to promote after a definite step in their careers (5, 8, 9, 20, 47–50). In a 10-year update (2010–2019) of American Board of Thoracic Surgery female cardiothoracic surgeons, while there was a decrease in those working as instructor or assistant professor with a correlating increase in associate professors, approximately the same proportion of women surgeons reported being a full professor as 10 years ago, suggesting potential stagnation at the associate professor rank (10). Given that the number of female physicians declines with ascending hierarchy or higher leadership levels, particular paucity of women in leadership positions is also important in terms of difficulty in identifying same-sex mentors due to the lack of women in surgical leadership (9, 10, 47). In an analysis of in the Accreditation Council for Graduate Medical Education database including 1,179 surgeons in 78 cardiothoracic surgery academic programs in the United States, only 9.6% of surgeons were women and they represent 4.5% (17 of 376) of full professors and 5% (11 of 195) of division chiefs in cardiothoracic surgery along with shorter career durations (12). Hence, career duration, including cumulative research productivity, is considered the key factor predicting full professor rank, potentially contributing to persistent sex-based disparities in academic cardiothoracic surgery (12).

Accordingly, there is a need for better understating the types and impacts of different gender discrimination experiences in the workplace and for implementing strategies to address gender inequities in the healthcare workplace to change cultures of discrimination such as staff education, clear anti-harassment policies and changes to academic promotion processes, faculty recruitment and retention (2, 45, 51). In a systematic review of 12 studies on organizational barriers to and facilitators for female surgeons’ career progression, the major organizational factors contributing to the lack of career progression for female surgeons were reported to include organizational culture (the rigid career structure supporting mainly the male surgeons and the male domination) and work family conflict of women (compelled to make family sacrifice with difficulty in securing a work-life balance in male-dominated surgical specialties) (17). Hence, potential areas of improvement that should be considered by policy makers and healthcare organizations to encourage women to enter the field include the development of more family-friendly working conditions allowing work-life balance for female surgeons with reduction of stigma regarding social roles (i.e., motherhood, housework) and provision of more flexible parental leave options with affordable childcare resources on hospital campuses, flexible career pathways with redistribution of access and opportunities (scholarship and mentorship) for women in thoracic surgery, the development of female surgical associations and the increased female leadership (9, 10, 17, 18, 47, 52).

### Limitations

Certain limitations to this study should be considered. First, the qualitative cross-sectional study design limits our ability to make causal inferences Second, the low response rate is another important limitation in terms of non-response bias and generalizability of our findings, given the likelihood of characteristics to differ between responder and non-responder groups. Clearly there is a selection bias noting the percentage of female respondents, and not that many surgeons responded either which limits the conclusions that can be drawn about the surgeons. This could indicate that those who identify more with this particular topic are more likely to respond. Third, lack of data on psychometric tools to assess the impact of gender discrimination on work performance, self-efficacy, emotional well-being is another limitation which otherwise would extend the knowledge achieved in the current study. Nonetheless our findings provide preliminary data on gender inequality and a framework for assessing attitudes and experience of gender discrimination by physicians practicing in chest diseases and thoracic surgery clinics.

## Conclusion

In conclusion, our findings revealed that exposure to gender discrimination in work life was more commonly expressed by female members of TTS who consider this to have negative impact on their career, as men are given priority in academic career promotion and employment. Female doctors were more likely to be pulmonologists rather than thoracic surgeons, while none of the female thoracic surgeons were professors and majority were working as a resident or specialist, emphasizing that women remain underrepresented in thoracic surgery, particularly in the academic rank of full professor and in leadership positions with inability to promote after a definite step in their careers. Female doctors, particularly those at younger ages, considered the discriminatory, negative and dissuasive attitudes of male physicians, as well as the traditional family and social roles and unwieldy maternity-leave policies as the main factors contributing to less likely preference of surgical branches by females, while male doctors considered higher success of men in specialties that require active physical strength and those with very long working hours and heavy shifts. Accordingly, changing standards and culture to appropriately address and prevent gender discrimination in traditionally male-dominated fields such as thoracic surgery, through a positive work environment allowing women surgeons putting in more time and effort toward work that appropriately translate into career advancement, leader position and job satisfaction, is critical to encourage female doctors to enter this field.

## Author’s note

This paper was presented in a poster session at the 28th International Congress of the European Respiratory Society which was held on September 15–19, 2018, in Paris, France.

## Data Availability

The original contributions presented in the study are included in the article/Supplementary material, further inquiries can be directed to the corresponding author.

## Group members of Turkish thoracic society women and lung diseases working group

Arzu Yorgancıoğlu (Celal Bayar University Faculty of Medicine, Aydin, Turkey); Birsen Ocaklı, Sinem Güngör, Hilal Altınöz, Elif Özarı Yıldırım, Tülin Sevim, Eylem Acartürk Tunçay, Cansel Atinkaya Öztürk, Ipek Özmen, Emine Aksoy (University Health Sciences Istanbul Sureyyapasa Chest Diseases and Thoracic Surgery Training and Research Hospital, Istanbul, Turkey);Yeşim Yiğiter Şenol, Başak Göktaş, Gülay Özbilim (Akdeniz University Faculty of Medicine, Antalya, Turkey); Yelda Varol, Gülru Polat, Serir Özkan(University of Health Sciences Dr. Suat Seren Chest Diseases and Thoracic Surgery Training and Research Hospital, Izmir, Turkey); Pınar Çelik (Celal Bayar University Faculty of Medicine, Aydin, Turkey); İlknur Genç Kuzuca, Serap Duru (University of Health Sciences Ankara Yildirim Beyazit Training and Research Hospital, Ankara, Turkey); Elif Yelda Niksarlıoğlu, Lütfiye Kılıç (University of Health Sciences Yedikule Chest Diseases and Thoracic Surgery Training and Research Hospital, Istanbul, Turkey); Ahmet Uğur Demir, Nagehan Emiralioğlu (Hacettepe University Faculty of Medicine, Ankara, Turkey); Göksel Altınışık, Neşe Dursunoğlu (Pamukkale University Istanbul Faculty of Medicine, Denizli, Turkey); Füsun Ö. Eyüboğlu, Gaye Ulubay (Baskent University Faculty of Medicine, Ankara, Turkey); Ezgi Özyılmaz, Ali Kocabaş (Cukurova University Faculty of Medicine, Adana, Turkey); Füsun Topçu (Dicle University Faculty of Medicine, Diyarbakir, Turkey), Nurdan Köktürk (Gazi University Faculty of Medicine, Ankara, Turkey); Yeşim Yasin (Acibadem University Faculty of Medicine, Istanbul, Turkey); Çağla Pınar Taştan (Bakırkoy Dr. Sadi Konuk Training and Research Hospital, Istanbul, Turkey); Esra Uzaslan (Uludag University Faculty of Medicine, Bursa, Turkey); Fatma Erboy (Bulent Ecevit University Faculty of Medicine, Zonguldak, Turkey); Bilun Gemicioğlu (Istanbul University Cerrahpasa Faculty of Medicine, Istanbul, Turkey); Bülent Karadağ (Marmara University Faculty of Medicine, Istanbul, Turkey); Özlem Erçen Diken (Hitit University Faculty of Medicine, Corum, Turkey); Seyhan Dülger (Bursa Yuksek Ihtisas Training and Research Hospital, Bursa, Turkey); Feride Taşkin Yılmaz (Cumhuriyet University Faculty of Medicine, Sivas, Turkey); Yasemin Söyler (Ankara Atatürk Chest Diseases and Thoracic Surgery Training and Research Hospital, Ankara); Hülya Yolaçan (Eskisehir Acibadem Hospital, Eskisehir, Turkey); Pınar Yıldız Gülhan (Duzce University Faculty of Medicine, Duzce, Turkey); Nursel Dikmen (Mustafa Kemal University Faculty of Medicine, Hatay, Turkey); Olcay Ayçiçek (Karadeniz Technical University Faculty of Medicine, Trabzon, Turkey); Şebnem Yosunkaya (Meram Faculty of Medicine, Konya, Turkey); Nursel Türkoğlu (Antalya Training and Research Hospital, Antalya, Turkey); Buğra Kerget (Ataturk University Faculty of Medicine, Erzurum, Turkey); Rabia E. Ünver (Orhangazi State Hospital, Bursa, Turkey); Münevver Mertsoylu Aydın (Kayseri State Hospital, Kayseri, Turkey); Aygül Güzel (Ondokuz Mayis University Faculty of Medicine, Samsun, Turkey); Adalet Demir (Istanbul University Istanbul Faculty of Medicine, Istanbul, Turkey); Füsun Yıldız (Girne University Faculty of Medicine, North Cyprus); Salih Topçu (Kocaeli University Faculty of Medicine, Kocaeli, Turkey); Pelin Duru Çetinkaya (Adana Numune Training and Research Hospital, Adana, Turkey); Pınar Pazarlı (Bilgi University Vocational School of Health Services, Istanbul, Turkey); Sebahat Genç (Mugla University Faculty of Medicine, Mugla, Turkey).
