# Supplementary material for: Attitudes toward and exposure to gender discrimination in work life by pulmonologists and thoracic surgeons: a questionnaire-based survey among Turkish thoracic society members
Source: Front Med (Lausanne). 2024 Nov 20;11:1463732. doi: 10.3389/fmed.2024.1463732 (PMC11614615; doi:10.3389/fmed.2024.1463732)
Supplement: Supplementary file 1 [file Data_Sheet_1.docx]

**ATTITUDES TOWARD AND EXPOSURE TO GENDER DISCRIMINATION IN WORK LIFE BY TURKISH THORACIC SOCIETY MEMBERS: QUESTIONNAIRE FORM**

*Thank you for agreeing to take this survey. The purpose of the survey is to investigate attitudes toward and exposure to gender discrimination in work life by Turkish Thoracic Society members. All of the answers you provide in this survey will be kept confidential.*

**PART I: SOCIODEMOGRAPHIC CHARACTERISTICS**

| **Age** |  | ….…. years |
| --- | --- | --- |
|  |  | *Please mark* |
| **Gender** |  |  |
| Male |  |  |
| Female |  |  |
| **Marital status** |  |  |
| Married |  |  |
| Single |  |  |
| Divorced |  |  |
| **Number of children** |  |  |
| 1 |  |  |
| 2 |  |  |
| ≥3 |  |  |
| **Partner’s occupation** |  |  |
| No partner |  |  |
| Unemployed |  |  |
| Physician |  |  |
| Other |  |  |
| **Hospital type** |  |  |
| University hospital |  |  |
| State hospital |  |  |
| Training and research hospital |  |  |
| **Specialty** |  |  |
| Pulmonologist |  |  |
| Thoracic surgeon |  |  |
| Other (resident, general practitioner, family physician) |  |  |
| **Academic title** |  |  |
| Resident |  |  |
| Specialist |  |  |
| Asst. Prof. |  |  |
| Assoc. Prof. |  |  |
| Prof. |  |  |
| Other |  |  |

**PART II–GENDER DISCRIMINATION IN WORK LIFE**

*Please indicate your agreement/disagreement to the below statements*

|  | | **Strongly disagree**  **(1)** | **Disagree**  **(2)** | **Neutral**  **(3)** | **Agree**  **(4)** | **Strongly agree**  **(5)** |
| --- | --- | --- | --- | --- | --- | --- |
| **General opinions and attitudes towards gender discrimination** | |  |  |  |  |  |
| 1 | The sex of physician should be a criterion to be considered during provider selection |  |  |  |  |  |
| 2 | Besides work in the workplace, housework is also an important burden for the female physician. |  |  |  |  |  |
| 3 | If a sacrifice is to be made between spouses for a career in working life, this task falls on woman. |  |  |  |  |  |
| 4 | Women physicians are exposed to more discrimination because of their clothes. |  |  |  |  |  |
| 5 | Social roles (motherhood, housework) are largely influential on the specialty choices of female physicians |  |  |  |  |  |
| 6 | Women physicians are more successful in passive jobs that require patience and punctuality. |  |  |  |  |  |
| 7 | Men are more successful in specialties that require active physical strength (surgery, etc.) |  |  |  |  |  |
| 8 | Specialties with very long working hours and heavy shifts are more suitable for men |  |  |  |  |  |
| 9 | Female physicians cannot succeed in surgical branches |  |  |  |  |  |
| 10 | Patients trust male doctors more and take them seriously |  |  |  |  |  |
| 11 | I think that the discriminatory, negative and dissuasive attitudes of male physicians played a role in the female physicians not choosing surgical branches. |  |  |  |  |  |
| 12 | Female physicians are more exposed to the disturbing behavior of the opposite sex in their professional life compared to male physicians due to their gender. |  |  |  |  |  |
| **Exposure to gender discrimination in work life** | |  |  |  |  |  |
| 1 | My gender had an impact on my choice of the department I am currently in |  |  |  |  |  |
| 2 | I have been exposed to gender discrimination throughout my career |  |  |  |  |  |
| 3 | While performing my profession, my gender negatively affected my working life |  |  |  |  |  |
| 4 | In the unit where I work, men are given priority in academic career promotion |  |  |  |  |  |
| 5 | In the unit where I work, work-sharing is based on the order of precedence regardless of the gender |  |  |  |  |  |
| 6 | In the unit where I work participation in decision making (getting opinions etc.) does not depend on gender |  |  |  |  |  |
| 7 | In the unit I work in, sexist expressions such as “try do a man’s job and “women’s reason” are used in conversations and discussions |  |  |  |  |  |
| 8 | The facilities of the department I work in (education, participation to congress etc.) are used without any gender discrimination |  |  |  |  |  |
| 9 | In the unit where I work, opinions of men rather than women are considered despite their same order of precedence |  |  |  |  |  |
| 10 | In the unit where I work, mostly male physicians are preferred due to the reasons such as pregnancy and breast-feeding permissions. |  |  |  |  |  |

Thank you very much for taking the time to complete our survey.
